# Origin of metamagnetism in skyrmion host Cu$_2$OSeO$_3$

**DOI:** 10.1038/s41598-022-20038-5

**Published:** 2022-09-24

**Authors:** Harish Chandr Chauhan, Birendra Kumar, Subhasis Ghosh

**Affiliations:** School of Physical Sciences, Jawaharlal Nehru University, New Delhi, 110067 India

**Keywords:** Condensed-matter physics, Physics

## Abstract

Skyrmion host chiral Cu$_2$OSeO$_3$ has attracted researchers due to several intriguing properties. Observation of metamagnetism in low-temperature and low-field makes the magnetic properties of Cu$_2$OSeO$_3$ more complex. Here, we present an investigation on metamagnetism in Cu$_2$OSeO$_3$ by analyzing its structural and magnetic properties. Study of magnetic properties reveal spin-flip of one of the Cu$^{2+}$ ions, embedded in square pyramidal CuO$_5$ polyhedra, due to the development of strain in low-temperature and low-field regime. The spin-flip is found to be the main reason for field-induced first-order metamagnetic transition. Magnetic phase diagram of Cu$_2$OSeO$_3$ has been constructed with the help of magnetization analyses. It is argued that the metamagnetic hysteretic field region may be low-temperature skyrmion phase with additional spiral and tilted-conical phases. A tricritical point has been observed in the phase diagram at which first-order metamagnetic hysteretic field range ceases to exist.

## Introduction

Skyrmion, a vortex like spin-texture, has been observed in various magnetic systems having different crystal symmetries^1–14^. Skyrmion host magnetic systems are good candidate for information carriers and magnetic memory devices^15–17^. Generally, skyrmions are observed in centrosymmetric and noncentrosymmetric magnetic materials in a small window of applied field and temperature^1,2,4,11^. In centrosymmetric systems, magnetic bubbles, which are topologically equivalent to skyrmion phase, emerge due to competition between symmetric exchange interaction (SEI) and dipole-dipole interaction^12,13,18,19^ under external magnetic field. So, it is important to study the nature of SEI, *i.e*, whether it is Heisenberg type, Ising type or some other type. In noncentrosymmetric magnetic systems^1–8^, skyrmions emerge due to competition between SEI and antisymetric-Dzyloshinskii-Moria interaction (DMI)^20,21^ under external magnetic field. Multiple magnetic phases, such as helical, conical, field polarized (FP) and fluctuation disordered (FD), also evolve with temperature under external magnetic field^1–8^. Skyrmion phase emerges in a small pocket of applied field and temperature. So, a general Hamiltonian, which is responsible for the formation of various magnetic phases, can be written as

1 $$\begin{aligned} \begin{aligned} \mathscr {H}=&\mathscr {H}_{SEI}+\mathscr {H}_{DMI}+\mathscr {H}_{d}+\mathscr {H}_{Ani}+\mathscr {H}_{Zeeman} \\ =&\sum _{ij}J_{ij}{} {\textbf {S}}_{i}.{\textbf {S}}_{J}+\sum _{ij}{} {\textbf {D}}_{ij}.({\textbf {S}}_{i}\times {\textbf {S}}_{j})+K_d\sum _{ij}\Bigg (3\frac{({\textbf {S}}_{i}.{\textbf {r}}_{ij})({\textbf {S}}_{j}.{\textbf {r}}_{ij})}{r^{5}_{ij}} - \frac{{\textbf {S}}_{i}.{\textbf {S}}_{j}}{r^{3}_{ij}}\Bigg )+\mathscr {H}_{Ani}-\mu _0{\textbf {H}}.{\textbf {M}}. \end{aligned} \end{aligned}$$

where $\mathscr {H}_{SEI}$, $\mathscr {H}_{DMI}$, $\mathscr {H}_{d}$, $\mathscr {H}_{Ani}$ and $\mathscr {H}_{Zeeman}$ are the Hamiltonian for SEI, DMI, dipole-dipole interaction^13,22,23^, anisotropic interaction and Zeeman interaction, respectively. $J_{ij}$ is the coefficient of exchange interaction, ${\textbf {D}}_{ij}$ is Dzyaloshinskii-Moriya vector and $K_d$ is the coefficient of dipole-dipole interaction. The dipolar energy is effective in low dimension, such as two-dimension (2D)^22^, thin-films^23^ and layered systems^13^. In bulk systems, dipolar energy is negligibly small^24^. That is why, in noncentrosymmetric chiral cubic systems, dipolar energy is generally neglected leading to $K_d=0$^25–27^. In centrosymmetric magnetic systems ${\textbf {D}}_{ij}=0$^21^. $\mathscr {H}_{Ani}$ is highly dependent on the chosen system which is why expression for $\mathscr {H}_{Ani}$ is not given^28–34^. In Cu$_2$OSeO$_3$, magnetocrystalline anisotropy, with easy axis along [111], plays key role in various physical phenomena^35–37^. The exchange coefficient $J_{ij}$ also varies with chosen materials. For example, the physics of MnSi and FeGe are governed by single value of *J* while the physics of Cu$_2$OSeO$_3$ is governed by using at least four values of *J* depending on the chosen Cu$^{2+}$ ions^25–27^. The magnetic phase diagram of several skyrmion host magnetic materials have been reported with the help of various techniques such as Lorentz transmission electron microscopy (LTEM)^4^, small-angle neutron scattering (SANS)^4^, specific heat^38^, magnetic susceptibility^38^ and magnetic isotherm^39,40^. A detailed investigation on different phases and phase transition between them has made it possible to explore the existence of various critical points such as Lifshitz point^40^, tricritical point^38,40^, and triple point^41^.

Cu$_2$OSeO$_3$ is the only known magnetic material in which the skyrmion phase has been observed in two different pockets of applied field and temperature^35,42–45^. High-temperature skyrmion (HTS) phase has been observed in the vicinity of $T_C(\simeq 59\,K)$, and the low-temperature skyrmion (LTS) phase has been observed in low-temperature regime ($T<<T_C$). As discussed above, the competition among SEI, DMI, magnetocrystalline anisotropy, thermal energy and Zeeman energy give rise to skyrmion and other phases. So, it is natural to ask–How skyrmions emerge at two different pockets of applied field and temperature? Interestingly, the properties of HTS and LTS are different^35,42–45^. The SANS results show−six-fold intensity pattern for HTS while ring plus six-fold intensity pattern for LTS phase^35,42,44^. The LTS phase has been observed only along [100] direction^44^ hinting the lowering of cubic symmetry due to some structural deformation, such as the emergence of magnetoelastic anisotropy which leads to completely different scenario of competing energies. So, the Hamiltonian (Eq. 1) should be different for HTS and LTS phases. Moreover, similar SANS result (ring plus six-fold intensity pattern) has been observed for FD phase in MnSi^46,47^. It has been investigated that the FD phase is thermally induced chiral fluctuations caused by first-order Brazovskii transition^48^. However, the explained physics for LTS is completely different from the physics for FD phase in spite of having almost similar SANS results. It is concluded that rings intensity pattern may arise from thermodynamically stable skyrmionic correlation, which coexist with spiral and tilted-conical phases^42^. Also, the sudden jump in the magnetization isotherm with hysteresis has been observed in Cu$_2$OSeO$_3$ at low-temperature ($T<<T_C$), and is known as metamagnetic transition^49^. More importantly, the LTS phase emerges in the metamagnetic hysteretic field range (has been discussed in detail later). Bannenberg *et al.*^42^ have explained the physics of different spin-textures with theoretical investigation by incorporating strength of cubic anisotropy. Here, the important question is− How does metamagnetic behavior appear in Cu$_2$OSeO$_3$? Thus, the observation of skyrmion in two different pockets of applied field and temperature, and several other novel magnetic phases, more importantly, the manipulation of skyrmions with an electric field^50^, necessitates the further investigation of metamagnetism in Cu$_2$OSeO$_3$^49^. Generally, the materials showing metamagnetism undergo first-order phase transition from the state of low magnetic moment to the state of the high magnetic moment in the presence of magnetic field^51–53^. Metamagnetism has been observed in various magnetic systems having different structural properties such as linear chain systems, two sublattice systems, four sublattice systems, garnets and mixed crystals^54^. Metamagnetic transitions have been observed in: (i) highly anisotropic systems due to local spin reversal, and (ii) isotropic or weak anisotropic systems due to the rotation of local spin directions^54^. Cu$_2$OSeO$_3$ has two types of CuO$_5$ polyhedra − square pyramidal and trigonal bipyramidal in the ratio of 3:1 − which leads to the formation of two sublattices^4^. As discussed above, formation of two sublattices causes the emergence of the magnetocrystalline anisotropy in the system with easy axis along [111]^35–37^. Thus, below $T_C$, the phase transition and the critical phenomena will be facilitated by SEI, DMI and anisotropies. Also, there should exist a tricritical point at which metamagnetic behavior disappears, leading to a continuous variation of magnetization. The physics of the metamagnetic properties of Cu$_2$OSeO$_3$ with associated tricritical point has not been explored yet.

Here, we present investigation on the origin of metamagnetism by incorporating structural and magnetic analyses of skyrmion host Cu$_2$OSeO$_3$. It is found that spin-flip of one of Cu$^{2+}$ ions (embedded with aquare pyramidal polyhedra) is the main reason for metamagnetism. The possible spin textures of metamagnetic hysteretic field range has been discussed comprehensively. A complete phase diagram of Cu$_2$OSeO$_3$ has been presented. A tricritical point, where first-order metamagnetic hysteretic field range ceases to exist and second-order helical to conical phase transition emerges, has been found.

## Experimental details

**Figure 1:**
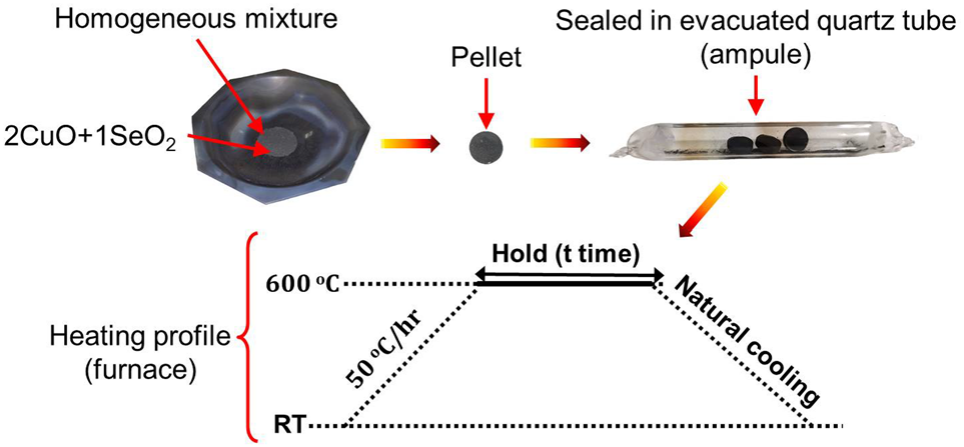
Schematic representation of the synthesis of Cu$_2$OSeO$_3$. First, homogeneous mixture of CuO and SeO$_2$ was taken in the molar ratio of 2:1, respectively. Then, pellets were made using hydraulic pressure and sealed in an evacuated quartz tube. The heating profile of the reaction was performed as the given schematic cycle. Intermediate grindings were performed to achieve single phase of the sample. RT: room temperature.

Cu$_2$OSeO$_3$ was grown using solid−state reaction method^40^. High purity CuO (Sigma Aldrich 99.999 %) and $\rm{SeO}_2$ (Sigma Aldrich 99.999 %) were taken in the molar ratio of 2:1, respectively. The mixture of CuO and $\rm{SeO}_2$ were grinded together for at least 8 hrs to obtain the homogeneous mixture. After making pellets of the homogeneous mixture, the sample was sealed in an evacuated quartz tube, which was then placed into a muffle furnace and annealed at 600 °C with heating rate of 50 °C/hr. The sample was held at 600 °C for 5 weeks. The intermediate grinding was performed for phase purity of the sample. Figure 1 represents experimental procedure used for the synthesis of Cu$_2$OSeO$_3$ sample. The X-ray diffraction (XRD) data of Cu$_2$OSeO$_3$ were collected using Rigaku Miniflex 600 X-Ray Diffractometer with Cu−K$_\alpha$ radiation. Phase purity of the sample was confirmed using Rietveld refinement of the XRD data^40^. High precision magnetic measurements were performed using physical properties measurement system. Field cooled (FC) and zero field cooled (ZFC) magnetization data were collected at 10, 20, 40 and 100 mT in warming mode. Four-quadrant field-dependent magnetization (M-H) isotherms were collected up to 250 mT at 2, 10, 20, 30, 40, 50, 57, 60, and 70 K. The step increment of the field was 1 mT. The magnetization data were recorded via following process: (i) first forward scan from 0 to 250 mT, (ii) first reverse scan from 250 to $-250$ mT, (iii) second forward scan from $-250$ to 250 mT, and (iv) second reverse scan from 250 to 0 mT. The analyses of Arrott-plots, susceptibility and phase diagram were carried out using the first-quadrant M-H isotherms of the second forward field scan.

## Results and discussion

### Structural analysis

**Figure 2:**
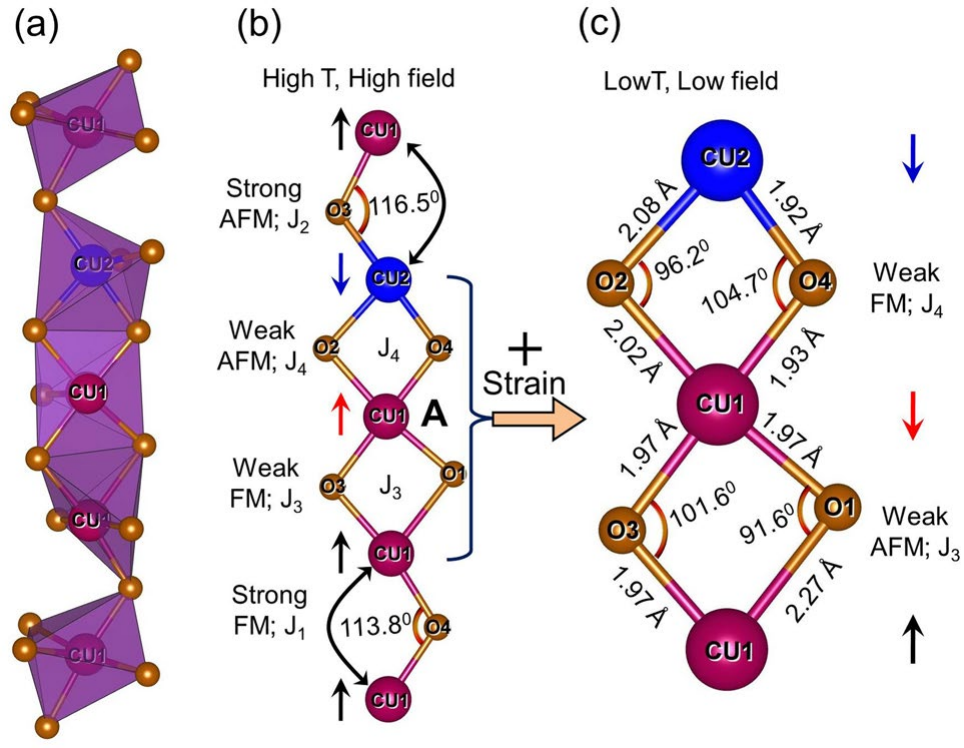
(**a**) Single lattice chain of CuO$_5$ polyhedra. Square pyramidal (around Cu1) and trigonal bipyramidal (around Cu2) CuO$_5$ polyhedra are present in the crystal structure in the ratio of 3:1. The lattice constant of the cubic crystal structure of Cu$_2$OSeO$_3$ is $a = 8.922$ Å. (**b**) Single lattice chain of Cu−O−Cu bonding. J$_1 = -3.693$ meV and J$_3 = -1.132$ meV are ferromagnetic superexchange couplings. J$_2 = 6.534$ meV and J$_4 = 0.900$ meV are antiferromagnetic superexchange couplings. These J values have been assigned from Ref.^25^. The directions of spins are shown schematically. (**c**) Edge sharing polyhedra showing weak AFM and weak FM. This is the most probable structure which can crossover from weak FM to weak AFM and vice versa with the development of magnetoelastic anisotropy which causes development of strain in the material.

The cubic crystal structure of Cu$_2$OSeO$_3$ with the lattice constant $a = 8.922$ Åwas estimated from the room temperature XRD analysis using Rietveld refinement^40^. Cu$_2$OSeO$_3$ lacks inversion symmetry and has 3-fold rotational symmetry along [111] direction. Here, square pyramidal (around Cu1) and trigonal bipyramidal (around Cu2) polyhedra are in the ratio of 3:1 as shown in Fig. 2a. The unequal ratio of Cu1 and Cu2 result in the formation of local ferrimagnetic ordering^4,41^. Figure 2b shows a simple lattice chain of Cu−O−Cu atoms estimated from the crystal structure. These lattice chains exhibit in the form of trigonal pyramid in which each lattice chain resides at the edges of the pyramid. In a single unit cell of Cu$_2$OSeO$_3$ ($a=8.922$ Å), each lattice chain consists of three Cu1 and one Cu2 atom. J$_1$ and J$_2$ are the superexchange couplings (Fig. 2b) between Cu atoms connected with corner-sharing CuO$_5$ polyhedra as shown in Fig. 2a. J$_3$ and J$_4$ are the superexchange couplings (Fig. 2b) between Cu atoms connected with edge-sharing CuO$_5$ polyhedra as shown in Fig. 2a. Zhang et al.^55^ suggested that the edge-sharing Cu−O−Cu atoms have weaker exchange interaction than the corner-sharing. This means J$_1$ and J$_2$ are stronger than J$_3$ and J$_4$. They have also indicated that the interaction between two identical atoms having different environment favors antiferromagnetic (AFM) exchange coupling while the interaction between two identical atoms having similar environment favor ferromagnetic (FM) exchange coupling as shown in Fig. 2b. Yang et al.^25^ have investigated the strengths of the superexchange couplings present in Cu$_2$OSeO$_3$. Following the above arguments and using the results of Yang et al.^25^, we can assign values to different superexchange couplings as J$_1 = -3.693$ meV, J$_2 = 6.534$ meV, J$_3 = -1.132$ meV, and J$_4 = 0.900$ meV. Other groups have also estimated the values of the exchange couplings^26,27^. The estimated values from other groups^26,27^ are slightly different but the nature of the exchange couplings is same. It has been reported that the edge-sharing superexchange coupling may alter its coupling behavior (from weak FM to weak AFM and vice versa)^56^. Further discussions are carried out in the coming sections about−How spin-flip may occur in Cu$_2$OSeO$_3$?

### Magnetic analysis

#### Temperature-dependent magnetization

**Figure 3:**
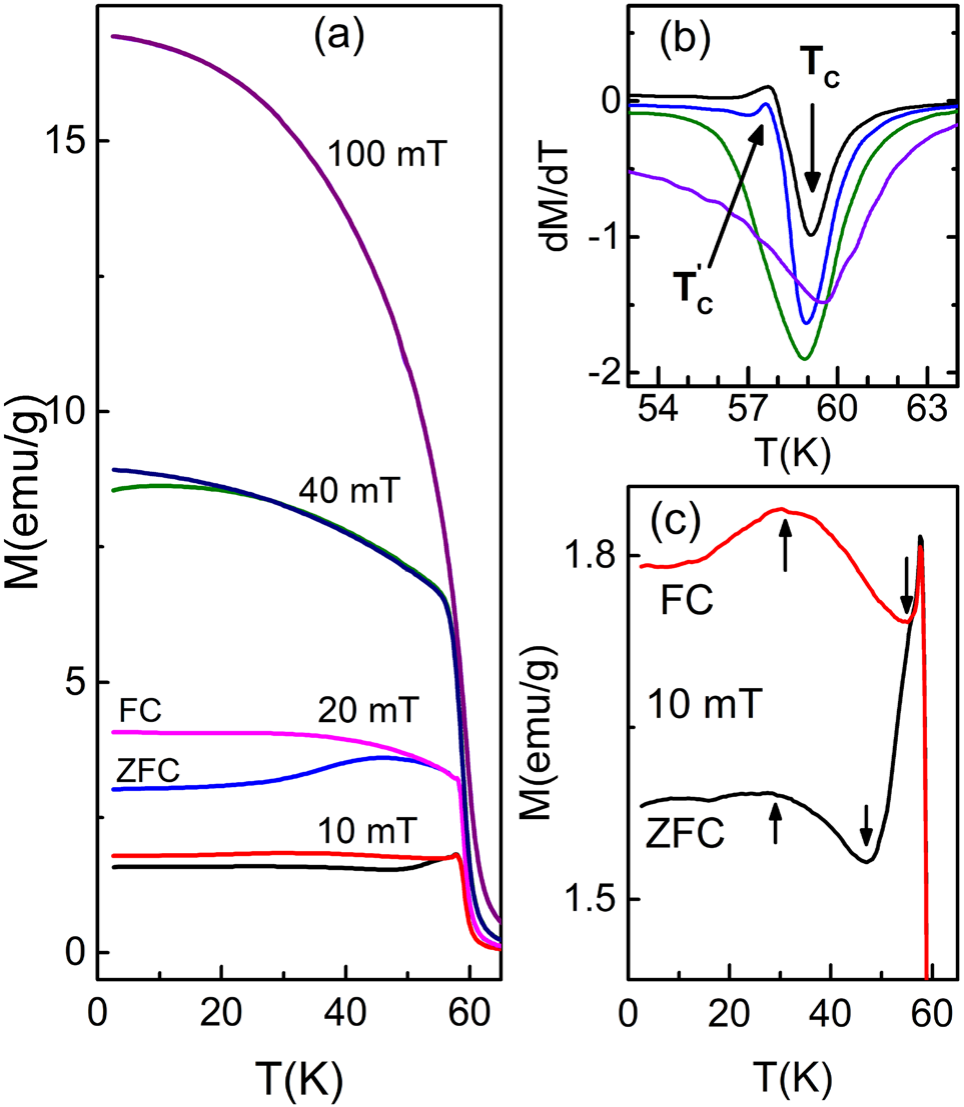
(**a**) FC and ZFC plots of the temperature-dependent magnetization (M-T) data taken at various applied magnetic fields as mentioned. (**b**) The first derivative of the ZFC curves. The minimum of the first derivative of the M-T plots give transition temperatures. An anomaly is observed in the derivatives of 10 and 20 mT. These transition points are consistent with the FD phase boundary. A complete field polarized ferrimagnetic ordering is observed at 100 mT. (**c**) Expanded view of the M-T curves taken at 10 mT.

Figure 3a shows the temperature-dependent magnetization (M-T) of Cu$_2$OSeO$_3$ taken at 10, 20, 40, and 100 mT. The minimum of the first derivative $\left( \displaystyle \frac{dM}{dT}\right)$ of the ZFC M-T isofields (Fig. 3b) yield $T_C$ $\approx$ 59.10, 58.95, 58.88, and 59.50 K at 10, 20, 40, and 100 mT, respectively. The $\displaystyle \frac{dM}{dT}$ at 10 and 20 mT show peak at 57.4 and 57.78 K (Fig. 3b), respectively. These transition points are consistent with the phase boundary of FD regime^40^, which is known as the precursor phase for the formation of helimagnetic spin textures in skyrmion host magnetic materials^38,40,57^. The bifurcation in the FC and ZFC M-T isofields are observed till 40 mT (Fig. 3a). Such a typical bifurcation occurs when the applied magnetic field is not enough to break and align the randomly bound magnetic moments in the direction of field. This effect is generally observed due to the presence of magnetocrystalline anisotropy in the system^35–37,58^. The strength of magnetocrystalline anisotropy decreases continuously with increasing temperature. In other words, the bifurcation width ($\Delta M$), which is the difference between the magnetic moment of FC and ZFC M-T isofields, should decrease with increasing temperature. In helimagnetic materials, domain wall trapping causes bifurcation in the M-T isofields and formation of hysteresis around origin in the M-H isotherms^59–61^. As observed in Fig. 3a, $\Delta M$ is less at 10 mT, and maximum at 20 mT. Also, $\Delta M$ increases with temperature at low applied field (Fig. 3c). Thus, the variation of $\Delta M$ is unusual in the context of magnetocrystalline anisotropy. This leads to the conclusion that there should exists some other anisotropy to compete with magnetocrystalline anisotropy. It seems that exchange anisotropy, which may cause spin-reversal, is locally effective at low-temperature and low-field in Cu$_2$OSeO$_3$.

The variation of FC and ZFC M-T taken at 10 mT is shown in Fig. 3c. In the vicinity of $T_C$, the cusp like behavior is observed due to the competitive effect of antisymmetric DMI and SEI. Similar kind of cusp has been observed in other chiral cubic B20 materials in which the physics is governed by single FM exchange coupling^62–64^. The M-T taken at 10 mT (Fig. 3c) shows unusual variation in comparison with the M-T of other skyrmionic hosts B20 materials^62–64^. In FC warming mode, the magnetic moment: (i) increases from 2 to 31 K, (ii) decreases from 31 to 55 K, (iii) further increases from 55 K to 57.7 K, and (iv) finally decreases to zero. This means the multiple superexchange couplings are causing such unusual variation^25^. The ZFC M-T taken at 10 mT (Fig. 3c) also shows similar unusual variation. In the absence of external magnetic field, magnetocrystalline anisotropy plays an important role by driving the magnetic moment along the easy axes. The magnetization at 10 mT shows following temperature-dependence in ZFC warming mode. The magnetic moment: (i) increases from 2 to 29 K, (ii) decreases from 29 to 47 K, (iii) further increases from 47 to 57.7 K, and (iv) finally decreases to zero. Below 31 K, the FC M-T looks alike the M-T for weak AFM material^65,66^. But, the variation of M-T does not reflect pure AFM variation due to presence of DMI, anisotropy energies and Zeeman energy. The above arguments lead to the conclusion that the crossover occurs from weak FM (in high temperature regime) to weak AFM (in low temperature regime) resulting small reduction in the magnetic moment. The crossover will occur only if one of the Cu spins get flipped which will cause change in effective anisotropies. The flipping of those spins, which are connected with weaker exchange couplings, is more favorable. So, the spins connected by J$_{3}$ and J$_{4}$ are most probable to get flipped resulting in the lowering of the magnetic moment. Mondal et al.^56^ have suggested that the change in the Cu−O−Cu bond angle may cause a crossover of the exchange coupling from FM to AFM and vice versa. The same crossover is possible in Cu$_2$OSeO$_3$ if strain develops at low temperature (see Fig. 2c). Bos et al.^49^ and Evans et al.^67^ have shown how strain indeed develops at low temperature in Cu$_2$OSeO$_3$. Thus, the strain developed will actually cause change in effective anisotropies. The observed cusp has disappeared in the M-T taken at high fields (see Fig. 3a) due to ineffectiveness of DMI^41^. The M-T taken at 20 mT also shows similar variation as discussed for the M-T taken at 10 mT. After a critical field ($\approx$ 40 mT), usual variation in the M-T isofields, which are the confirmation of the FP ferrimagnetic ordering, have been observed. The M-H isotherms have been further analyzed to find out the critical-field and critical-temperature responsible for spin-flip.

#### Field-dependent magnetization

**Figure 4:**
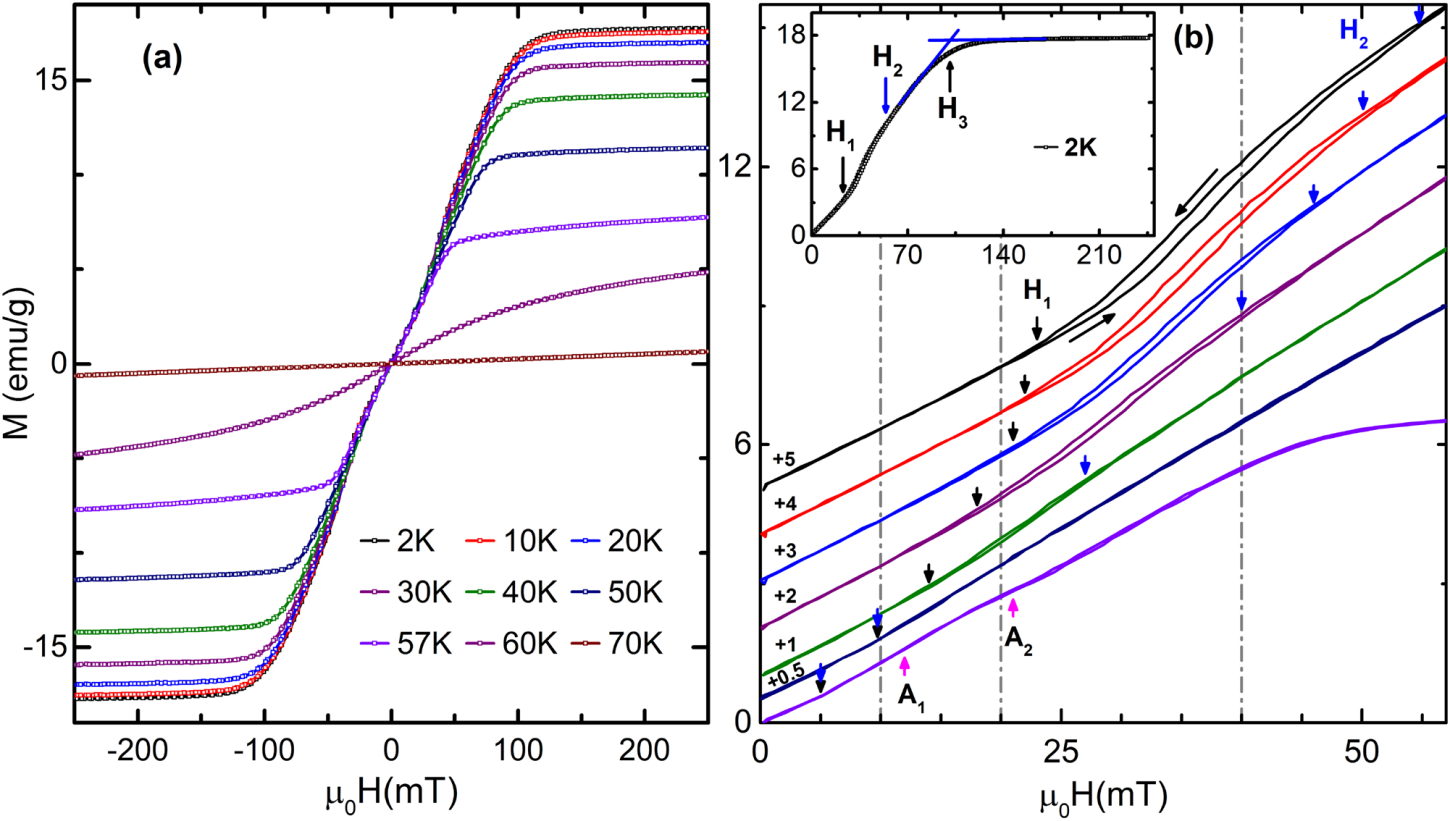
(**a**) M-H isotherms measured in the step mode up to 250 mT with increment step of 1 mT taken at various temperatures as mentioned. An unusual anomaly of magnetization variation is visible. (**b**) Expanded view of (**a**) to see the clear variation of M-H isotherms. The M-H isotherms have been shifted vertically as mentioned. The first forward scan (0–250 mT) isotherms is not shown due to substantial overlap with the M-H isotherms taken during second forward scan ($-250$ to 250 mT). There is no magnetic hysteresis around the origin. The metamagnetic hysteresis is observable from 2 to 40 K in the intermediate applied field range H$_1$ to H$_2$. The M-H data taken at 57 K shows step-like variation, which is related to skyrmion formation in Cu$_2$OSeO$_3$. A$_1$ and A$_2$ represent the skyrmion phase boundary. The inset of (**b**) represents the critical field H$_3$, above which field-induced ferrimagnetic phase has been observed.

The saturation magnetization of Cu$_2$OSeO$_3$ is $\approx$ 0.53 $\mu _B$/Cu$^{2+}$ ion^49,68^, which is approximately half of the magnetic moment of S $=$ 1/2 spin state (1 $\mu _B$) for each Cu$^{2+}$ ion. This confirms ferrimagnetic spin arrangement [3(up):1(down)] in Cu$_2$OSeO$_3$^49^ in the FP region. Figure 4 shows the M-H isotherms taken up to 250 mT at various temperatures. The linear variation of magnetic moment is observed at small applied field up to H$_1$ (Fig. 4b). This is same as the variation of the magnetic moment observed for helical phase^8^. The magnetic moment increases abruptly from H$_1$ to H$_2$ (Fig. 4b) forming hysteresis in between. This hysteretic field range decreases with increasing temperature and disappears above 40 K. The transition from the low magnetic moment (below H$_1$) to high magnetic moment (above H$_2$) with hysteresis between H$_1$ and H$_2$ is the characteristic feature of first-order metamagnetic transition^51–54,69,70^. The sudden jump in the magnetic moment is possible only if some Cu spins are flipped in the direction of the applied field. Based on the strength of superexchange couplings, those Cu spins, which are connected with J$_3$ and J$_4$, are more likely to be flipped. This is possible only if strain develops in the low-temperature regime causing change in the effective anisotropies. As discussed before, the saturation magnetic moment is $\approx$0.53 $\mu _B$/Cu$^{2+}$ due to 3(up):1(down) spin configuration above H$_2$. This implies that spins are antiferromagnetically coupled due to spin-flip below H$_1$ and 50 K. The schematic representation of spins is shown in Fig. 2c. The above points lead to the conclusion that Hamiltonian should be different below H$_1$ and 50 K. All these analyses indicate that spin at position **A** (Fig. 2b) is most likely to be flipped. All the above discussions are consistent with the M-T isofields as shown in Fig. 3. The phase between H$_2$ and H$_3$ is the conical phase^8^. The magnetic properties of first-order phase transition^71^ indicate that the metamagnetic hysteretic field region may be a mixed phase consisting of both helical and conical spin textures or, some other new phase. In analogy to Ref.^71^, a small fraction of conical spin textures will start appearing after H$_1$, and will increase with increasing field till H$_2$. Below H$_2$, small fraction of the helical phase will remain while conical phase fraction will be dominating. Indeed, SANS results show coexistence of LTS phase with spiral and tilted-conical phases^42^. Similar SANS result has been observed for FD phase due to thermally induced chiral fluctuation^46,47^. In the analogy, field-induced continuous spin-flip may cause inhomogeneity in LTS phase yielding ring intensity pattern along with six-fold intensity pattern in metamagnetic hysteretic field range. After H$_2$, conical phase will appear till H$_3$ as shown in the inset of Fig. 4b. Metamagnetic hysteretic phase (and hence metamagnetic transition) is observed below 50 K (see Fig. 4b). Second-order phase transition has been observed^40^ from helical to conical phase above 50 K. So the point (50 K, 9 mT) should be the tricritical point at which metamagnetic hysteretic field range ends, and direct helical to conical phase transitions starts. The M-H plot of Cu$_2$OSeO$_3$ at 57 K shows the emergence of field-induced HTS phase in the conical phase region^4,8,39,40^.

#### Arrott-plot and susceptibility

**Figure 5:**
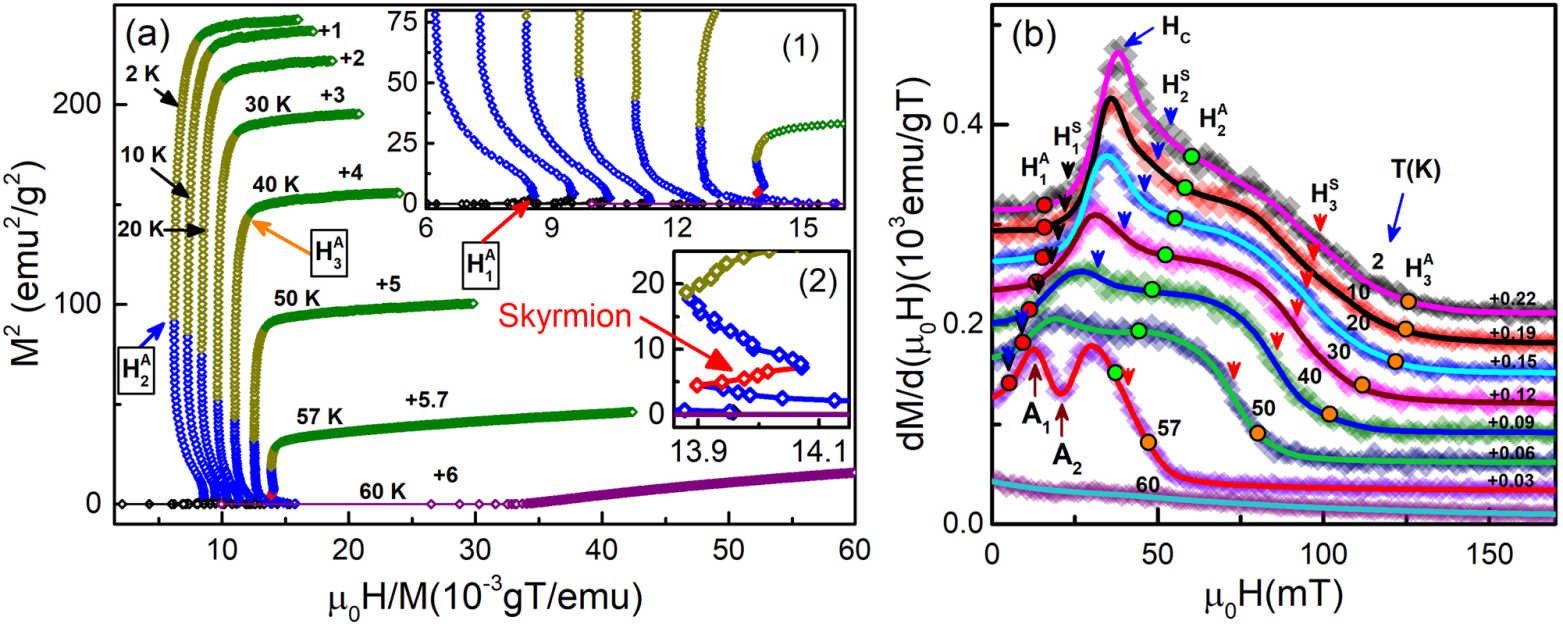
(**a**) Arrott-plots obtained from the first quadrant M-H isotherms recorded during second forward scan from $-250$ to 250 mT. Various positive and negative slope regions represent multiple phases observed in Cu$_2$OSeO$_3$. Inset shows the expanded view of the Arrott-plots. (**b**) Variation of magnetic susceptibility of Cu$_2$OSeO$_3$ estimated from the first derivative of the first quadrant M-H isotherms recorded during second forward scan from $-250$ to 250 mT. Symbols represent experimental data and solid lines are average fit to the experimental data. The phase below H$_1^S$ represents helical phase. Hysteretic phase region is observed between H$_1^S$ and H$_2^S$. Above H$_2^S$ and up to H$_3^S$, conical phase formation is estimated. Above H$_3^S$, field-induced ferrimagnetic phase formation occurs. Flat or almost zero susceptibility value of 60 K curve indicates the paramagnetic phase region.

Arrott-plot and susceptibility analyses have been further investigated to estimate the phase boundaries connecting different phases present in Cu$_2$OSeO$_3$. The Arrott relation^72^ is represented as

2 $$\begin{aligned} M^2=A\left( \frac{\mu _0H}{M}\right) +B, \end{aligned}$$

where $A=\displaystyle \frac{1}{b}$ and $B=-\displaystyle \frac{a}{b}$ are constants. Arrott relation^72^ is widely used to determine $T_C$^40^ and order of phase transition using Bannerjee’s criteria^73^. Figure 5a shows the Arrott-plots obtained using M-H isotherms. As, it can be seen [inset (1) of Fig. 5a] that there exist multiple positive and negative slope regions. Arrott-plots show following properties.

(i) Positive slopes below H$_1^A$.
(ii) Negative slopes from H$_1^A$ to H$_2^A$.
(iii) An intermediate positive slope region has been observed between A$_1$ and A$_2$ in the negative slope regions (H$_1^A$ to H$_2^A$) of the Arrott-plot taken at 57 K as shown in the inset(2) of Fig. 5a. The phase between A$_1$ and A$_2$ is consistent with observed HTS phase.
(iv) Positive slopes above H$_2^A$.

The higher field positive slope regions (above H$_2^A$) can be divided further into two parts− slow varying slope regions (almost vertical lines in Fig. 5a) from H$_2^A$ to H$_3^A$, and almost constant slope regions above H$_3^A$. Now, let us first examine the susceptibility plots before going into detailed analysis of different phases appearing in Cu$_2$OSeO$_3$. The magnetic susceptibility plot (Fig. 5b), obtained from the first derivative of the M-H isotherms give a clear picture of various transitions. H$_1^S$, H$_2^S$ and H$_3^S$ are the inflection points on the susceptibility curves below 50 K. The phase boundaries determined from M-H (Fig. 4b) and susceptibility (Fig. 5b) are consistent. Below H$_1^S$, susceptibility varies linearly, which is consistent with the linear variation of M-H isotherms below H$_1$ as shown in Fig. 4. On comparison with SANS^4^ and LTEM results, the following observation can be made.

(i) Helical phase^1–8^ is below H$_1^S$.
(ii) Conical phase is from H$_1^S$ to H$_3^S$ having intermediate metamagnetic hysteretic field range from H$_1^S$ to H$_2^S$ in low temperature regime. H$_C$ is the critical field where susceptibility is observed to be maximum. The susceptibility pattern from H$_1^S$ to H$_2^S$ is similar to the susceptibility for LTS phase^43^, which has been confirmed by SANS results^42,44,45^.
(iii) Dip in the susceptibility has been observed from A$_1$ to A$_2$ in the conical phase region. The phase between A$_1$ and A$_2$ is consistent with the HTS phase^42–45^.
(iv) Field induced ferrimagnetic phase appears above H$_3^S$.

The phase boundaries, constructed using Arrott-plots and susceptibility isotherms, are deviating more in the low temperature regime. H$_1^A$ and H$_1^S$ are almost overlapping in the whole temperature regime. It can be seen that H$_2^A$ is appearing in the conical phase region. With increasing temperature H$_2^A$ and H$_3^S$ get closer to each other. It seems that conical phase is stable till H$_3^S$ and may have different properties below and above H$_2^A$. After H$_3^S$, conical phase becomes unstable leading to formation of field induced ferrimagnetic phase having effective DMI till H$_3^A$. After H$_3^A$, complete ordered ferrimagnetic phase can be observed with negligibly small DMI^41^. So, the field-region from H$_2^A$ to H$_3^A$ may have mixed spin-textures of conical and field-induced-ferrimagnetic phases. And, spin-textures of conical and field-polarized-ferrimagnetic phases may have equal contribution at H$_3^S$.

### Phase diagram

**Figure 6:**
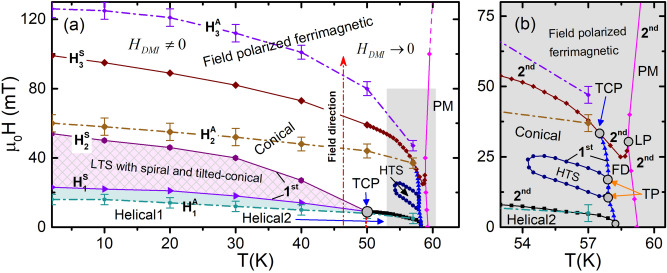
(**a**) Magnetic phase diagram of Cu$_2$OSeO$_3$ constructed with the help of FC-ZFC M-T isofields, first quadrant M-H isotherms recorded during second forward scan from $-250$ to 250 mT, Arrott-plots and susceptibility analyses. H$_1^A$, H$_2^A$ and H$_3^A$ are the phase boundaries constructed using Arrott-plot (Fig. 5a) analyses. H$_1^S$, H$_2^S$ and H$_3^S$ are the phase boundaries constructed using magnetic susceptibility (Fig. 5b) analyses. The paramagnetic phase boundary has been constructed using the M-T isofields as depicted in Fig. 3. The HTS phase boundary has been determined using Arrott-plot and susceptibility analyses. Helical phase is below H$_1^S$. LTS phase with added spiral and tilted conical phase is between H$_1^S$ and H$_2^S$. Conical phase is from H$_2^S$ to H$_3^S$. HTS phase has been observed in the vicinity of $T_C$ in conical phase region. FIeld-induced ferrimagnetic phase appears above H$_3^S$. Here, H$_1^A$ is almost same as H$_1^S$. H$_2^A$ represents the critical fields around which Arrott-plots change the slope from negative to positive. H$_3^A$ represents the critical fields above which DMI becomes ineffective. (**b**) Expanded view of the shaded (light-gray) region of (**a**). Detailed analysis of the shaded region has been done somewhere else^40^. Helical2, conical, HTS, FP ferrimagnetic, PM and FD phases are observed in the vicinity of $T_C$. A tricritical point (TCP) is observed at (57.5 K, 33 mT) where first-order FD to conical phase transition transform into second-order conical to field polarized ferrimagnetic phase. Lifshitz point (LP) is observed at (58.8 K, 30 mT) where ordered (FP ferrimagnetic), disordered (PM) and incommensurate (FD) phases meet tangentially. Two triple points (TPs) are observed at [(57.88 K, 17 mT) and (57.90 K, 10.50 mT)] where first-order FD, HTS and conical phases meet. HTS: high-temperature skyrmion, LTS: low-temperature skyrmion, FP: field polarized, FD: fluctuation disordered.

Figure 6 represents magnetic phase diagram of Cu$_2$OSeO$_3$ constructed using the above analyses. We have shown that spin-flip caused by the development of strain (refer to Fig. 2) may lead to different Hamiltonian below H$_2^S$ and below 50 K. Below H$_1^S$, spins try to align antiferromagnetically, but, due to presence of other competing interactions (as discussed above), the antiferromagnetically coupled spins may also yield helical spin-textures. So, it is quite possible to have two kinds of helical spin textures^45^: (i) helical1 below 50 K, and (ii) helical2 above 50 K. Due to spiral spin texture, the two helical phases may have identical properties. The similar property may not be observed in the M-T isofields due to almost similar spin texture in helical1 and helical2 phases. Metamagnetic phase transition occurs from H$_1^S$ to H$_2^S$ yielding metamagnetic hysteretic field range. The spins of metamagnetic hysteretic field range may form helical or conical or mixed spin texture^71^. On comparison with previous reports^35,42–45^, it is found that the LTS phase emerges in the field range over which metamagnetic hysteresis has been observed. Due to discontinuous spin-flip, ring intensity pattern may be observed in SANS. This ring intensity pattern may be the measure of coexistence of other spin-textures, such as spiral and tilted-conical, in LTS phase^42^. In analogy with FD phase^46,47^, the metamagnetic hysteretic field range may have field-induced spin-fluctuation. After H$_2^S$, the weakly bound Cu1 spins get flipped (Fig. 2) completely, and conical phase emerges due to presence of other competing energies. Thus, below H$_2^S$ and 50 K, the physics of Cu$_2$OSeO$_3$ will be governed by J$_1$ (strong FM), J$_2$ (strong AFM), J$_3$ (weak AFM), and J$_4$ (weak FM). Above H$_2^S$ and 50 K, the physics of Cu$_2$OSeO$_3$ will be governed by J$_1$ (strong FM), J$_2$ (strong AFM), J$_3$ (weak FM), and J$_4$ (weak AFM). Below H$_2^A$, Arrott-plots show negative slopes. So, it can be inferred that the conical phase may have different properties below and above H$_2^A$. The conical phase continuously transform into field polarized ferrimagnetic phase around H$_3^S$. It is possible that DMI may become ineffective above H$_3^S$ due to Zeeman energy and spin reorientation. And, DMI may become negligibly small (or zero) above H$_3^A$ leading to saturation magnetization^41^ as shown in Fig. 4. HTS phase has been observed in the conical phase in a small window of applied field and temperature^1–8,35,42–45^. The physics of the shaded region (53–61 K) has been explained comprehensively in previous reports^40,41^. A tricritical point (50 K, 9 mT) has been observed where metamagnetic hysteretic field range ceases to exist.

## Conclusion

Investigation of the unusual variation of the magnetic moment of the M-T curves confirms the flipping of spins due to the development of strain which causes change in effective anisotropies at low temperature. The crossover from weak FM to weak AFM causes first-order metamagnetic transition leading to the formation of metamagnetic hysteretic field region between helical and conical spin textures. LTS phase with added spiral and tilted-conical phases may have been observed in the metamagnetic hysteretic field range. Arrott-plots analyses confirm that there may exist conical phases having different properties. Also, same (positive or negative) slope regions of Arrott-plots may represent different phases if the slopes vary with different rates. The point (∼ 50 K, ∼ 9 mT) leads to the tricritical point where first-order metamagnetic hysteretic phase ceases to exist and second-order helical to conical phase transition starts. Finally, a magnetic phase diagram of Cu$_2$OSeO$_3$ has been constructed using the above analyses. Further investigations are required to explore the physics of different properties of LTS phase when compared with HTS phase.

## Data Availability

The data that support the findings of this study are available from the corresponding author upon reasonable request.
